# Microchimerism and pregnancy complications with placental dysfunction

**DOI:** 10.1007/s00281-025-01045-w

**Published:** 2025-03-11

**Authors:** Daniel Pitz Jacobsen, Heidi E. Fjeldstad, Maria B. Olsen, Meryam Sugulle, Anne Cathrine Staff

**Affiliations:** 1 Faculty of Medicine, University of Oslo, Oslo, Norway; 2 Division of Obstetrics and Gynaecology, Oslo University Hospital, Oslo, Norway; 3 Research Institute of Internal Medicine, Oslo University Hospital, Oslo, Norway; 4 Division of Obstetrics and Gynaecology, Oslo University Hospital, Oslo University Hospital, Kirkeveien 166, Box 4956, Oslo, Nydalen, Oslo, 0450, 0424 PO Norway

**Keywords:** Angiogenic markers, Mesenchymal stem cells, Microchimerism, Placental dysfunction, Tolerization, Pregnancy

## Abstract

Cells cross the placenta during pregnancy, resulting in proliferation of semiallogeneic cells in the mother and fetus decades later. This phenomenon, termed microchimerism, is documented across mammalian species, implying an evolutionary benefit. Still, short- and long-term effects remain uncertain. Here, we review the dynamics of microchimerism of fetal, maternal, and mother of the proband origin in relation to increasing gestational age and pregnancy complications associated with placental dysfunction including preeclampsia, fetal growth restriction, preterm labor, recurrent miscarriage, and diabetes. We use the two-stage model of preeclampsia as a framework. We recently published a series of papers independently linking increased fetal microchimerism to markers of placental dysfunction (stage 1), severe maternal hypertension (stage 2) and poor glucose control. Placental dysfunction may influence the intrinsic properties of fetal stem cells. Mesenchymal and hematopoietic stem cells isolated from cord blood during preeclampsia display reduced proliferative potential in vitro. Moreover, preeclampsia is shown to disrupt paracrine signaling in mesenchymal stem cells of the umbilical cord. Undesired properties in cells transferred to the mother could have profound negative effects on maternal health. Finally, recent studies indicate that microchimerism is involved in inducing maternal-fetal tolerance. Disruption of this process is associated with pregnancy complications. Long term, the persistence of microchimerism is necessary to sustain specific regulatory T cell populations in mice. This likely plays a role in the proband’s future pregnancies and long-term maternal and offspring health. Current evidence indicates that advancements in our understanding of microchimerism could be instrumental in promoting reproductive and long-term health.

**Supplementary Information:**

The online version contains supplementary material available at 10.1007/s00281-025-01045-w.

## Introduction

**Fig. 1:**
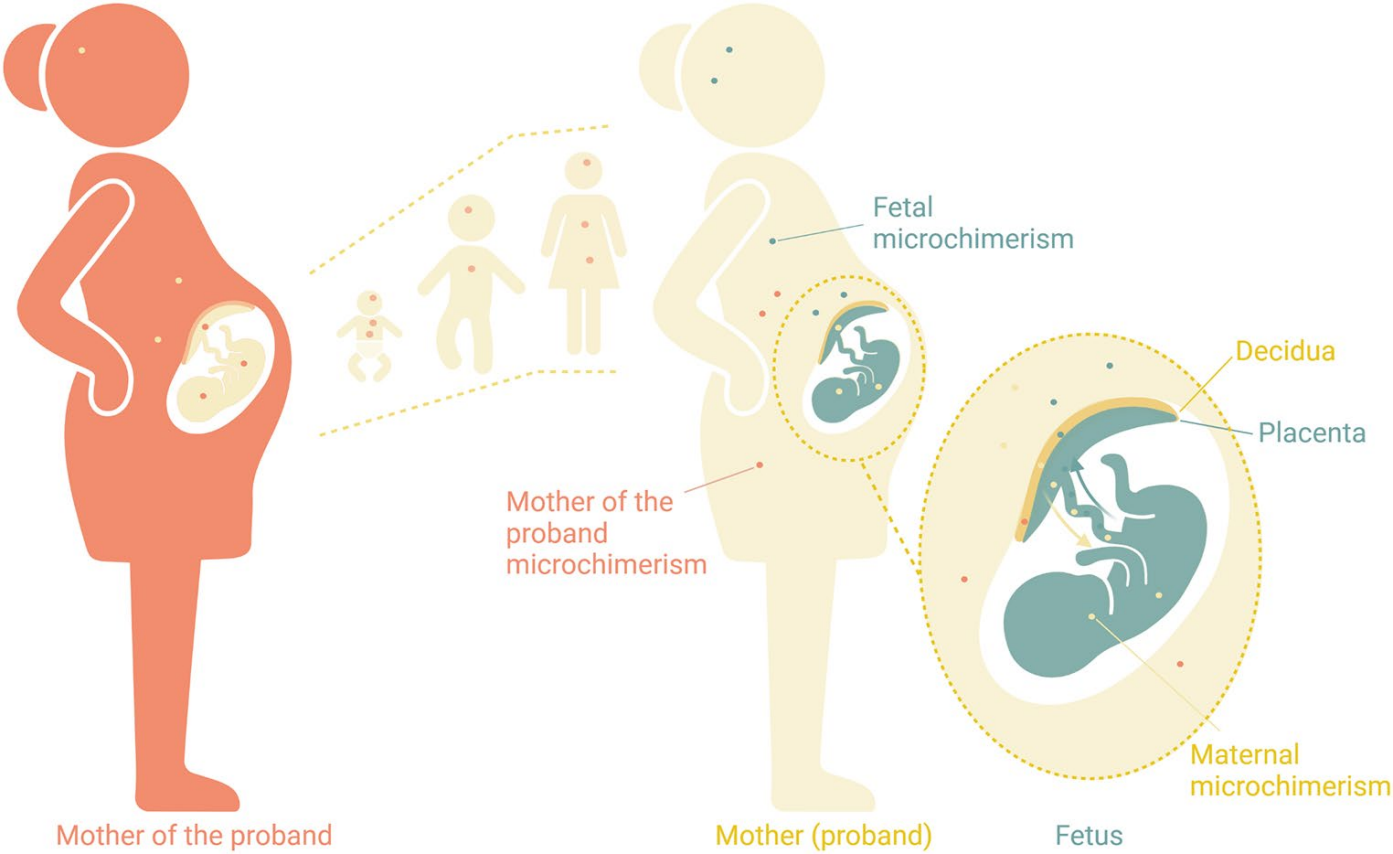
Illustration of cell transfer across generations. Cells are exchanged between the mother of the proband (orange) and the proband (yellow) in utero. Mother of the proband microchimerism remains in the proband into adulthood. During pregnancy, the proband aquires fetal microchimerism (green). Meanwhile the fetus aquires maternal microchimerism (yellow). Created with BioRender.com

Microchimerism denotes the presence of a relatively small proportion of allogeneic cells residing within an individual [1]. This phenomenon arises naturally during gestation, when bi-directional transfer of cells across the placental barrier gives rise to fetal microchimerism in maternal systems and maternal microchimerism in fetal systems [2] (Fig. 1). Some of these migrating cells have the ability to proliferate and differentiate within the host and have been observed decades after parturition. In women, cells possessing a Y-chromosome, of presumed fetal origin, have been detected in peripheral blood 27 years postpartum [1] and in the heart 24 years postpartum [3], exemplifying persistent fetal microchimerism in the mother. Microchimerism of both maternal and fetal origin has also been detected in various other tissues where the foreign cells adopt local cell phenotypes [4–6]. A child of any sex may harbor maternal microchimerism into adulthood. In addition, when a genetically female person becomes pregnant, the “proband” (i.e. the pregnant woman) potentially harbors cells from her own mother termed “mother of the proband” microchimerism [7]. She will also acquire cells from the fetus, termed fetal microchimerism, whilst the fetus simultaneously acquires “maternal microchimerism” from the proband (Fig. 1). In research, microchimeric cells are detected by targeting alleles not present in the host’s genotype. In fetal microchimerism, paternal alleles inherited by the fetus and not possessed by the proband, known as “inherited paternal alleles”, are detected in maternal blood or tissues. For example in the case of a male fetus, a genetic sequence found on the Y-chromosome would be an appropriate target for detection in maternal blood or tissues. In maternal microchimerism, maternal genetic sequences not inherited by the offspring, known as “non-inherited maternal alleles”, may be targeted in order to detect maternal cells in offspring blood or tissues.

Maternal and fetal microchimerism are found across mammalian species, implying an evolutionary benefit [8]. Indeed, recent findings suggest that microchimerism may play a role in placental trophoblast function [9] and immune tolerance during gestation [10, 11], thereby theoretically improving pregnancy outcomes and granting cross-generational reproductive fitness. Even though microchimeric cells are few in numbers, extracellular vesicles and other signaling modalities give these cells tremendous potential to affect pregnancy health, and their longevity gives them the potential to impact long-term health as well. The notion that fetal microchimerism provides long-term benefit to the mother is supported by several animal studies demonstrating fetal cell migration to sites of injury, contributing to angiogenesis, inflammation and tissue restoration [12–15] consistent with the demonstration of fetal-origin keratinocytes in human cesarean section scar tissue [5]. The notion that maternal microchimerism contributes to tissue regeneration in the offspring as well is supported by a study on type 1 diabetes reporting maternal microchimerism with a functional islet β-cell phenotype [6].

On the other hand, microchimerism may under certain circumstances have detrimental effects during pregnancy and beyond (as reviewed in [16]), potentially contributing to the links demonstrated between pregnancy complications like preeclampsia and chronic illnesses down the road, including maternal cardiovascular disease [17–19] and autoimmunity [20]. The observation that circulating cells of fetal origin are detected in higher numbers in women with systemic sclerosis [21] and scleroderma [22] has led researchers to propose that semiallogeneic fetal-origin cells contribute to autoimmune disease, either as targets of maternal immune cells or effector cells [23]. This could partially explain why autoimmune disorders overwhelmingly affect women. Interestingly, while male-origin cells were detected in the kidney samples of 64% of women with lupus nephritis, in contrast to none in the controls, the presence of these cells was associated with less severe forms of glomerulonephritis [24]. Whether microchimerism in this context acts as a driver of a distinct type of autoimmunity or as a beneficial reparative pathway remains to be elucidated. As there are certain features shared between autoimmune diseases and preeclampsia, it has been proposed that fetal microchimerism contributes to preeclampsia development [25]. In the sections below, we discuss in detail the positive correlation between fetal microchimerism and the severity of both placental dysfunction [26–28] and maternal severe hypertension [28]. Mother of the proband microchimerism has, on the other hand, been proposed to have a protective effect against preeclampsia [29]. We and others have proposed physiological and pathophysiological roles for fetal and maternal microchimerism extending to other placental syndromes as well [30]. A causal link between microchimerism (fetal or maternal) has, however, not yet been established.

In the present review, we start by summarizing the various pregnancy complications associated with placental dysfunction. We then discuss how microchimerism features in pregnancy may relate to pregnancy complications associated with placental dysfunction using the two-stage model of preeclampsia as a framework. We go on to discuss the possibility that the ultimate effects of microchimerism may depend on the intrinsic properties of the microchimeric cells, which in turn may depend on the microenvironment in the placenta and the maternal-fetal interface, as well as on fetal-maternal histocompatibility and tolerization.

## Preeclampsia and other pregnancy complications with placental dysfunction

### Preeclampsia: placental dysfunction and systemic maternal inflammation

**Fig. 2:**
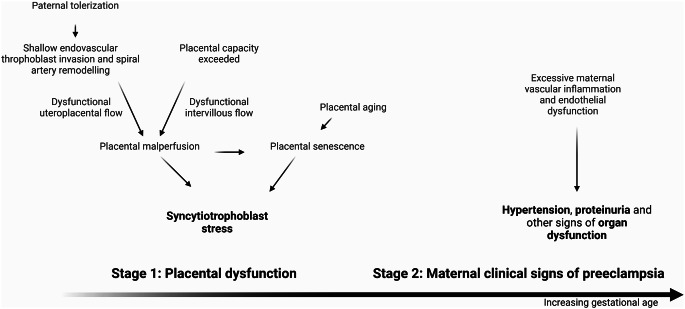
The two-stage model of preeclampsia. Multiple pathways can lead to placental dysfunction, which constitutes the first stage of preeclampsia development. Shallow trophoblast invasion and the subsequent poor spiral artery remodeling leads to abrasive, pulsatile uteroplacental blood flow, in turn resulting in placental oxidative stress, endoplasmic reticulum stress and activation of the unfolded protein response. Likewise, cellular placental stress can also arise later on in pregnancy, due to restricted intrauterine space, resulting in intervillous congestion and placental hypoxia. Lastly, placental senescence, either accelerated by other factors or occurring “naturally”, may also contribute to syncytiotrophoblast stress The subsequent dysregulated release of proinflammatory factors to the maternal vasculature, including increased release of antiangiogenic proteins, represents a link to the second stage. The second stage constitutes the ensuing maternal vascular inflammation and endothelial dysfunction resulting in the clinical manifestation of the disease. Created with BioRender.com

Preeclampsia is a hypertensive multi-system pregnancy disorder [31], and a leading cause of pregnancy mortality and morbidity worldwide for the mother and fetus. In addition, preeclampsia confers a substantially increased risk for premature maternal cardiovascular disease [17]. The two-stage model of preeclampsia may be used to describe its pathophysiology (Fig. 2). In this model, placental dysfunction constitutes the first stage of preeclampsia development [32, 33]. The subsequent dysregulated release of proinflammatory factors to the maternal vasculature, including increased release of antiangiogenic proteins, represents a link to the second stage. The second stage constitutes the ensuing maternal vascular inflammation and endothelial dysfunction resulting in the clinical manifestation of the disease (i.e. new-onset hypertension arising ≥ 20 weeks’, combined with either new-onset proteinuria or the other signs of maternal end-organ dysfunction and/or fetal growth restriction) [31]. More recently, acknowledging the essential role of placental dysfunction and the ensuing antiangiogenic state, biomarkers have been added to the preeclampsia definitions in international guidelines [34].

### Two pathways to placental dysfunction

Multiple pathways can lead to the first stage placental dysfunction described in the two-stage model [32, 33]. One pathway gives rise to placental dysfunction early in pregnancy and tends to lead to early-onset preeclampsia [33]. Fetal extravillous trophoblasts invade the maternal decidua basalis (the endometrium underlying the placenta), where they together with maternal decidual immune cells contribute to uteroplacental spiral artery remodeling, causing loss of arterial wall smooth muscle cells [35]. This vital process of placentation transforms the maternal arteries into flaccid conductors, facilitating constant, optimal uteroplacental blood supply [36] and is dependent upon maternal-fetal immune interactions [35]. Shallow trophoblast invasion and the subsequent poor spiral artery remodeling leads to abrasive, pulsatile uteroplacental blood flow, in turn resulting in placental oxidative stress, endoplasmic reticulum stress and activation of the unfolded protein response [37]. Under these conditions, the placental secretome shifts towards an anti-angiogenic profile by upregulating soluble fms-like tyrosine kinase-1 (sFlt-1) [38], an extracellular decoy receptor for placental growth factor (PlGF). Likewise, cellular placental stress arising later on in pregnancy, and thereby promoting development of late-onset preeclampsia, also results in anti-angiogenic imbalance of these factors in maternal circulation [33]. This can occur due to restricted intrauterine space, such as in pregnancies with large placentas (e.g. in multiples and at post-term), resulting in intervillous congestion and placental hypoxia [33]. The total and relative quantities of sFlt-1 and PlGF in maternal circulation correlate with histological placental characteristics [39] and reflect the degree of placental stress and dysfunction [32, 40].

### Placental cellular senescence

Placental stress and dysfunction is thought to relate in part or fully to accelerated placental senescence [32]. In fact, morphological studies of ageing placentas from healthy post-term pregnancies show an increased frequency of syncytial knots akin to what is seen in placentas from preeclamptic pregnancies [39]. These syncytial knots display evidence of loss of transcriptional activity and oxidative damage [41]. Of relevance, post-date pregnancies (defined as those that deliver after their due date, defined in said study as 40 + 2 weeks’ gestation) also had a more anti-angiogenic profile than those who delivered at term prior to or at their due date [42]. This in turn correlated with the histological findings [39]. Put together, these results support a link between physiological placental senescence and increasing placental dysfunction that could tip the mother over into a preeclamptic state depending on the timing of placental dysfunction onset and responsiveness of her cardiovascular system to excessive pro-inflammatory signals. It has been proposed that all pregnancies would inevitably eventually become complicated by preeclampsia if not naturally “rescued” by labor and delivery of the placenta [43].

### Other pregnancy complications linked to placental dysfunction

Gestational hypertension without any other accompanying maternal or fetal adverse clinical features is also associated with anti-angiogenic imbalance, although to a lesser extent [44]. Likewise, fetal growth restriction can occur in isolation (or as part of the preeclampsia syndrome) as a result of placental dysfunction [45], and is thus also linked to dysregulated release of sFlt-1 and PlGF into maternal circulation [46]. Fetal growth restriction resulting from poor placentation at the onset of pregnancy often results in low birthweight percentiles, whereas growth restriction resulting from placental dysfunction arising later in pregnancy may not [47]. In fact, a fair portion of the neonates with late-onset placental dysfunction are born large for gestational age [47].

Interestingly, a subset of pregnancies complicated by spontaneous *preterm* labor and delivery (occurring prior to 37 + 0 weeks’ gestation) are also associated with a maternal anti-angiogenic shift [48, 49], supporting a link to placental senescence and dysfunction in the underlying pathophysiology. Likewise, one of the causes of recurrent miscarriage is also thought to relate to dysregulated maternal-fetal immune interactions and poor placentation [35]. Placental abruption is another pregnancy complication thought to result from early-onset placental dysfunction due to poor trophoblast invasion [50]. However, an anti-angiogenic imbalance in maternal circulation has only been shown to correlate with placental abruption co-occurring with hypertensive disorders of pregnancy [51]. Finally, diabetes mellitus during pregnancy is also associated with placental dysfunction [52], possibly due to disturbed blood-oxygen supply to the uteroplacental unit resulting from excess amounts of glycosylated hemoglobin with increased oxygen affinity [53].

### Microchimerism dynamics in pregnancy and across placental syndromes

The mechanistic details of cross-placental cell transfer are currently unknown [30, 54]. Placental perfusion from the maternal compartment has demonstrated that lymphocytes may adhere to maternal vessel walls and enter the fetal villi and fetal circulation [55, 56]. Likewise, perfusion of the fetal vessels with fluorescently labelled mesenchymal stem cells resulted in cell migration across fetal vessel walls [57]. These findings suggest that certain cell types may actively migrate across the placenta by adhering to endothelial cell-surface molecules and crossing into the surrounding tissue [54]. On the other hand, damage to fetal capillaries and the progressive thinning of the placental barrier with increasing gestational age, often exasperated during placental dysfunction, may facilitate passive leakage of cells across the placenta as well. Accordingly, the detection of fetal erythroblasts is elevated during preeclampsia [58]. More studies are needed to elucidate which mechanisms are at play and how particular disorders of pregnancy might influence the mechanisms of fetal-maternal cell trafficking.

### Levels of complexity to microchimerism research during pregnancy

Microchimerism in pregnancy and pregnancy complications is a broad topic as it encompasses both fetal microchimerism in the mother and maternal microchimerism in the fetus arising as a result of bi-directional transplacental exchange. In addition, maternal microchimerism in the pregnant woman, known as “mother of the proband microchimerism”, acquired many years prior during her own fetal development, may be present (Fig. 1). Furthermore, the amounts and cellular phenotypes of the various types of microchimerism may vary with gestational age in clinically healthy pregnancies as well as in various pregnancy complications. In Table 1 we summarize microchimerism findings across gestational age and the pregnancy complications related to placental dysfunction that are outlined above.

**Table 1:** Overview of the dynamics of different types of microchimerism in healthy and complicated pregnancies

	Fetal microchimerism		Mother of the proband microchimerism		Maternal microchimerism	
Advancing gestational age (healthy)	↓ trophoblasts ↓erythroblasts ↑ PBMC ↑ buffy coat	[54] [53] [51] [55]	↑ PBMC	[26]		
Preeclampsia	↑ trophoblasts ↑ leukocytes ↑erythroblasts ↑ PBMC ↑ B-cells, NK-cells	[57] [58–60] [61] [62] [66]	↓ PBMC	[26]	– PBMC	[62]
Fetal growth restriction	↑erythroblasts ↑ buffy coat	[67] [64]				
Placental dysfunction / Anti-angiogenic shift	↑erythroblasts ↑ buffy coat	[63] [55, 64, 65]				
Severe hypertension	↑ buffy coat	[64]				
Recurrent miscarriage			↓ PBMC ↑ granulocytes	[68] [7]		
Preterm labor	– PBMC	[70]			↑ PBMC	[70]
Diabetes mellitus / poor glucose control	↑ erythroblasts ↑ buffy coat	[71] [65]				

### Fetal microchimerism dynamics in clinically healthy pregnancies

In clinically healthy pregnancies, the analysis of maternal peripheral blood mononuclear cells demonstrates a positive association between gestational age and both fetal [59] and mother of the proband microchimerism [29]. This could reflect increased cell leakage due to physiological placental barrier thinning, observed to occur toward the end of gestation [60]. On the other hand, maternal blood levels of fetal-origin erythroblasts [61] and cells of trophoblast origin in the venous return from the uterus [62] appear to be highest early on in pregnancy. This suggests a more controlled transfer mechanism might be at play or that fetal-origin cells engrafted in the mother may differentiate and proliferate at different gestational ages. In our study of maternal buffy coat, the “white blood cell compartment”, from normotensive pregnancies between 37 and up to 42 weeks’ gestation, we found an association between not only fetal microchimerism and increasing gestational age, but also between fetal microchimerism and a maternal anti-angiogenic shift [26]. The latter association remained significant after adjustment for gestational age, suggesting that the degree of placental cellular senescence and thereby placental dysfunction, rather than chronological placental age, impacts the degree of transplacental transfer or proliferation of fetal white blood cells in maternal circulation.

### Maternal microchimerism dynamics in clinically healthy pregnancies

As for the cross-placental transfer of maternal cells relative to gestational age, the sampling of umbilical cord blood across trimesters in healthy pregnancies is precluded by the ethical contraindication to sampling umbilical cord blood in utero without a medical indication. However, degree of HLA compatibility between the mother and neonate has been identified as a predictive marker of maternal microchimerism in cord blood [63]. It would be interesting to study whether maternal microchimerism acquisition correlates with placental dysfunction at time of delivery, measured by increased sFlt-1 and reduced PlGF in maternal circulation, as we have shown for fetal microchimerism [26].

### Fetal microchimerism in preeclampsia

Preeclampsia is probably the placental syndrome most comprehensively studied with respect to microchimerism, starting with the discovery of trophoblast-like cells in the lungs of women who had died of eclampsia in the 1800s by Georg Schmorl [64]. Karyotyping studies of leukocytes in maternal blood likewise showed an increase in male cells in women with preeclampsia [65–67]. More recently, studies have demonstrated an increase in both fetal-origin erythrocytes and peripheral blood mononuclear cells isolated from maternal blood in women with preeclampsia relative to gestational age matched controls [58, 68]. Interestingly, one group demonstrated an increase in fetal cells in pregnant women who only later went on to develop preeclampsia relative to women who remained healthy, arguing that damage to the placenta must be sufficient to cause increased fetal microchimerism in preeclampsia [69]. This latter finding fits with evidence from our recent studies as well. Specifically we recently demonstrated a correlation between maternal anti-angiogenic shift and increased fetal microchimerism not only in new-onset hypertensive disorders of pregnancy (including preeclampsia and gestational hypertension) [28] and pregestational diabetes mellitus [27], but also in normotensive term pregnancies [26] respectively, supporting our hypothesis that placental dysfunction in itself is enough to cause increased fetal microchimerism.

On the other hand, a recent study revealed that compared to healthy pregnancy, preeclampsia was associated with higher numbers of immune cells of fetal origin in maternal circulation [70]. It is possible that the observed elevation in subsets of fetal cells is either due to increased selective placental transfer of certain cell types, or due to increased proliferation and differentiation of certain fetal cells sequestered amongst maternal immune cells in response to generalized inflammation. The latter is in line with our findings in hypertensive disorders of pregnancy, demonstrating a distinct association between severe hypertension and fetal microchimerism, supporting that endothelial dysfunction and vascular inflammation lead to increased fetal microchimerism [28]. Put together, these findings have lead us to argue that *both* stages of preeclampsia development, rather than one or the other, likely contribute to and/or are impacted by the increased amounts of fetal microchimerism observed in preeclampsia, a topic further reviewed elsewhere [30].

### Maternal microchimerism in preeclampsia

The amount of maternal microchimerism in *fetal cord blood* during preeclampsia appears to be the same as in healthy term pregnancies [71]. The authors of said study proposed that this might reflect a difference in the transplacental trafficking of maternal versus fetal cells. One caveat is that the cases and controls were not gestational age matched as women with preeclampsia were delivered at earlier gestational ages.

### Fetal microchimerism and fetal growth abnormalities

As described above in the section on placental syndromes, one of the clinical features that may occur either in isolation or as part of the preeclampsia syndrome is fetal growth restriction [33]. We and others found an increase in fetal cells in maternal circulation during preeclamspia accompanied by fetal growth restriction, relative to those with only preeclampsia in the buffy coat compartment [28] and amongst erythrocytes respectively [72]. However, it appears that this association might be attributable to the underlying placental dysfunction; in our analysis of women with hypertensive disorders of pregnancy, statistical adjustment for placental confounders eliminated the association between fetal microchimerism and fetal growth restriction, defined in our study as birth weight < 3rd percentile according to fetal sex and gestational age at delivery [28]. Similarly, the presence of fetal growth abnormalities, defined by us as birth weight < 10th percentile or > 90th percentile, in a case series of women with gestational diabetes mellitus, was associated with increased fetal microchimerism in maternal buffy coat [27]. This association also diminished with adjustment for confounders related to placental dysfunction, thus further supporting the hypothesis that placental dysfunction is linked to increased fetal microchimerism in maternal circulation.

### Mother of the proband microchimerism in preeclampsia and recurrent miscarriage

Both early-onset preeclampsia and recurrent miscarriage are thought to relate to poor placentation. Mother of the proband microchimerism, specifically amongst peripheral blood mononuclear cells, is decreased in preeclampsia, lending support to the hypothesis that the degree of circulating maternal microchimerism from the proband’s own mother may be a marker reflecting healthy placentation and adaptation to pregnancy [29]. This hypothesis is also in line with an exploratory study suggesting that mother of the proband microchimerism in the peripheral blood mononuclear cell layer may be decreased prior to conception in cases of recurrent miscarriage when compared to healthy controls [73]. The same authors subsequently showed that mother of the proband microchimerism in the proband’s granulocyte layer was more frequently detectable in women who miscarried, particularly in those experiencing recurrent miscarriage [7]. These findings led them to hypothesize further that “maternal microchimerism (in the mother) when upregulated amongst specific adaptive immune cell subsets may facilitate implantation, while a shift toward its presence in innate cell types could interfere with implantation” [7].

When viewed in light of the two-stage model of preeclampsia, the above findings suggest that stage 1 placental dysfunction caused by poor placentation is tied to diminished mother of the proband microchimerism. The finding that mother of the proband microchimerism increases with increasing gestational age suggests that it does not, however, correlate with stage 1 placental dysfunction resulting from physiological placental cellular senescence. Furthermore, it is interesting to speculate as to whether circulating mother of the proband microchimerism has a protective effect against stage 2 endothelial dysfunction and vascular inflammation.

### Maternal and fetal microchimerism in preterm labor

A study of preterm labor showed that maternal microchimerism was significantly *increased* in the peripheral blood mononuclear cell layer of cord blood from preterm infants when compared to healthy term controls [74]. Fetal microchimerism in the maternal peripheral blood mononuclear cell layer remained unchanged [74]. Preterm labor thereby exhibits a pattern of fetal and maternal cell exchange opposite that described in preeclampsia [68]. In addition, the authors of the aforementioned study found a strong association between maternal microchimerism and the presence of fetal central memory T cells in cord blood and that the cord blood of preterm infants had higher amounts of inflammatory cytokines and greater activation of dendritic cells [74]. The combination of inflammatory cytokines, early activation of dendritic cells, and increase in maternal microchimerism was suggested to result in fetal effector T cell priming against maternal antigens. Finally, the authors concluded that “fetal inflammation and rejection of maternal antigens can contribute to the signalling cascade that promotes uterine contractility and that aberrant fetal immune responses should be considered in the pathogenesis of preterm labor” [74]. Such a link between maternal microchimerism in cord blood and premature parturition has, however, not been directly investigated.

### Fetal microchimerism in diabetes mellitus

As described above, diabetes mellitus during pregnancy is associated with placental dysfunction and it therefore stands to reason that the degree of cellular exchange between mother and fetus may be impacted. Accordingly, a study from 2004 demonstrated an elevated median percentage of cells with Y signals (of presumed fetal origin) in maternal blood in diabetic pregnancies compared to normal controls [75]. One of the mechanisms by which placental dysfunction is thought to arise in diabetic pregnancies is by reduced oxygen delivery to the placenta by the glycosylation of maternal haemoglobin [53]. We therefore examined whether the degree of glucose control in both pregestational and gestational diabetes mellitus respectively would impact the amount of fetal microchimerism in maternal buffy coat [27]. In both case series, we found a significant association between poor glucose control and increased fetal microchimerism. Interestingly, in the pregestational diabetes cohort, we found an association between increased fetal microchimerism and anti-angiogenic shift in sFlt-1 that remained significant after adjusting for poor glucose control [27]. This suggests that factors in addition to poor glucose control contribute to placental dysfunction and increased fetal microchimerism in this setting. In gestational diabetes mellitus on the other hand, we did not find an association between increased anti-angiogenic shift and increased fetal microchimerism. This could be due to low statistical power as this particular case series was small (*n* = 45 in the gestational diabetes mellitus cohort versus *n* = 77 in the pregestational diabetes mellitus cohort), or it could indicate that in gestational diabetes mellitus there are other drivers of fetal microchimerism increase that take precedence. One such driving factor could be excessive maternal vascular inflammation. It is tempting to speculate that fetal microchimerism may play a role in the epidemiological link between gestational diabetes mellitus and increased risk of preeclampsia.

### Mesenchymal stem cells, trophoblast invasion and fetal microchimerism

Mesenchymal stem cells of fetal origin are detected in maternal circulation during pregnancy [76], as well as in maternal immune tissue postpartum [77]. The presence of fetal “stem cells” in fetal microchimerism seemingly fits with the findings that fetal microchimerism can persist long term in multiple tissues. However, when it comes to mesenchymal stem cells, their function may not be entirely of a *stem cell* nature. The concept of mesenchymal stem cells was first introduced by Caplan in his seminal 1991 publication, where he proposed a role for them as a new therapeutic technology in regenerative medicine [78]. Decades of research into their biological function has revealed that these cells are not only multipotent, but also hold regenerative potential through rejuvenating paracrine signalling [79]. In fact, Caplan recently wrote a paper urging that mesenchymal stem cells be renamed to medicinal signalling cells [80]– keeping the commonly used abbreviation MSC– arguing that they do not behave as stem cells in vivo [81]. Instead, he argues, their main function is secretory and it is rather the resident stem cells that are responsible for reconstructing and repairing injured or dysfunctional tissues in response to trophic factors released by the mesenchymal stem cells [82]. As it turns out, this quality may have profound implications for pregnancy complications both locally in the placenta, corresponding to the first stage in the two-stage model, and systemically in the mother, corresponding to the second stage.

**Fig. 3:**
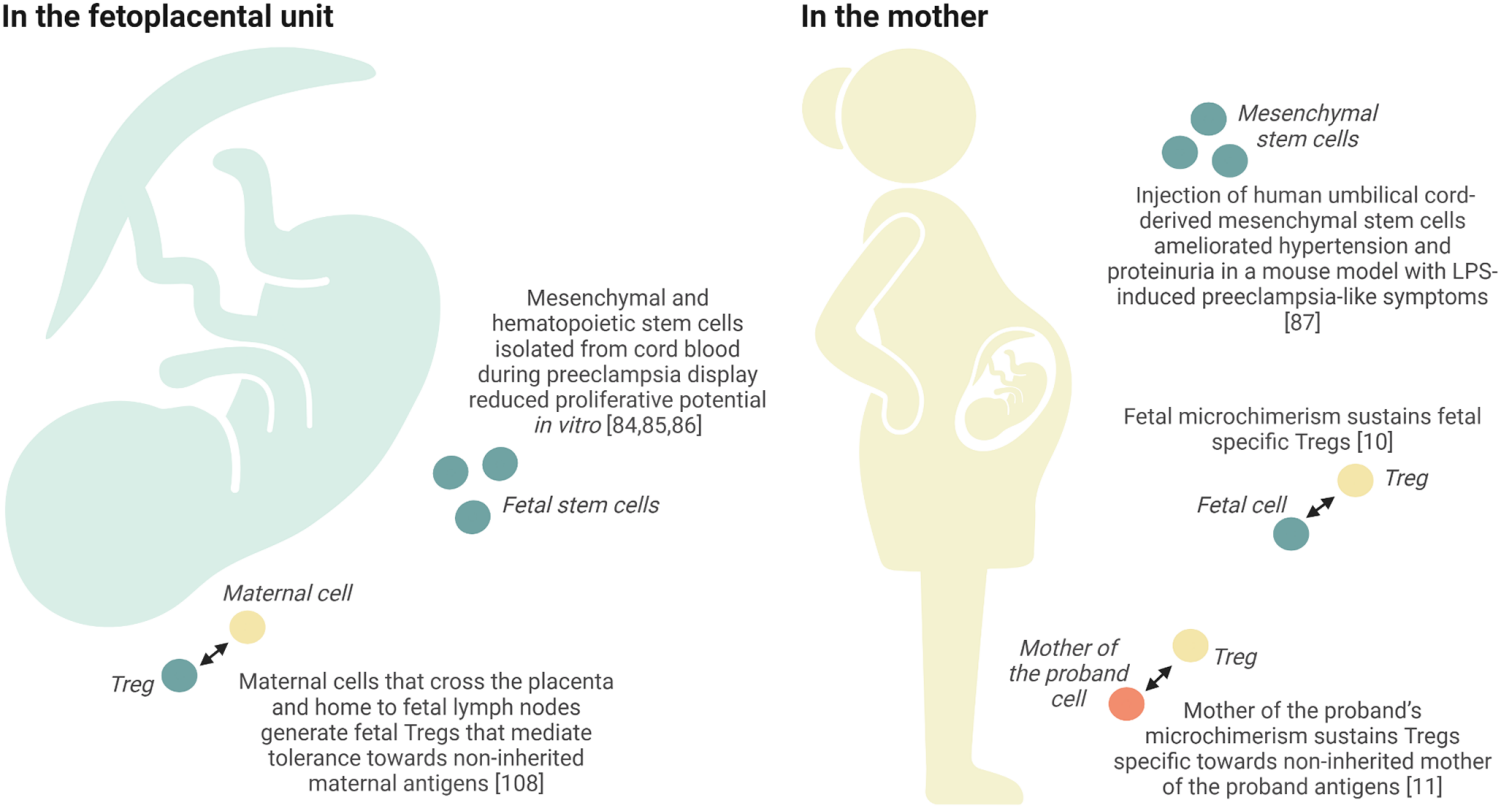
Possible roles for microchimerism in repair and tolerance. A graphical summary of findings relating to fetal cell function and the potential role of microchimerism (mother of the proband, maternal or fetal) in the development and maintenance of tolerance, according to whether they may occur in the fetoplacental unit or in the mother. Created with BioRender.com

In the context of “stage 1” placental dysfunction, chorionic plate-derived mesenchymal stem cells have been shown to significantly increase the invasive properties of trophoblasts in vitro, an essential function for physiologic pregnancy development [83]. Interestingly, simply applying supernatant from umbilical cord-derived mesenchymal stem cells increased trophoblast migration [84], demonstrating that the positive effect is indeed likely due to paracrine signaling. The authors of both these papers postulate that mesenchymal stem cells could act as treatment for placental dysfunction and placenta-related diseases [83, 84]. As to mesenchymal stem cell involvement in the pathophysiology of placental syndromes like preeclampsia and fetal growth restriction, others have suggested that detrimental cross-talk between dysfunctional fetal mesenchymal stem cells and trophoblasts may be involved [85]. They show that mesenchymal stem cells isolated from the umbilical cord (Wharton’s jelly) during preeclampsia or intrauterine growth restriction had reduced viability in vitro (Fig. 3) [85]. Moreover, these cells released high levels of NO, and had dampened positive effects on cultured trophoblast cells. Similar findings have been observed in mesenchymal stem cells isolated from cord blood [86]. During preeclampsia, these cells present with a senescent phenotype and increased endogenous superoxide production. Interestingly, hematopoietic stem cells from cord blood display similar characteristics during preeclampsia, with reduced colony-forming capacity, causing the authors to conclude that cord blood banking is not suitable for mothers with preeclampsia [87]. Whether the fetal cells that are transferred to the mother during preeclampsia also possess these undesired properties is currently unknown, but it is plausible considering they likely originate from the same populations that were examined in the studies mentioned above [85–87]. This process could thereby also impact “stage 2” endothelial dysfunction and inflammation in the mother.

Of relevance in the context of the second stage of preeclampsia development, functional mesenchymal stem cells of fetal origin may have a positive systemic effect on maternal tissues, to a degree mitigating the cardiovascular burden of pregnancy. Injection of human umbilical cord-derived mesenchymal stem cells ameliorated hypertension and proteinuria in a mouse model with LPS-induced preeclampsia-like symptoms (Fig. 3) [88]. In these mice, mesenchymal stem cell treatment also reversed vascular and placental inflammation. Due to the mouse model used, this effect is likely attributable to systemic effects of human umbilical cord-derived mesenchymal stem cells, rather than effects on extravillous trophoblast invasiveness. Interestingly, we recently reported a positive correlation between severe maternal hypertension and fetal microchimerism presence and quantity in women with preeclampsia [28]. It is tempting to speculate that this is due to increased natural recruitment of fetal mesenchymal stem cells in response to maternal endothelial dysfunction and vascular inflammation [28]. This might instigate a self-perpetuating negative cycle as recruitment of dysfunctional or senescent fetal mesenchymal stem cells could further exacerbate endothelial dysfunction in women with preeclampsia. However, the characterization of fetal microchimeric cell types and senescence markers in this setting remain to be investigated.

### Microchimerism and tolerance

Maternal immune tolerance towards the fetus is essential for a healthy pregnancy. This is consistent with studies showing that a shorter period of sexual cohabitation prior to conception (less than 3 months), and thus less exposure to paternal antigens to build tolerance [89], is associated with increased risk of developing preeclampsia [90]. Moreover, this acquired tolerance seems to persist over time as subsequent pregnancies with the same partner carry significantly reduced risk of infant mortality and other adverse pregnancy outcomes [91]. The same pattern is documented in mice, where repeated mating with the same paternal alloantigens grants resilience towards fetal wastage [10, 92]. Extravillous trophoblasts do not have strong immunogenicity, but they do express polymorphic HLA-C on the cell surface [93] and HLA-DR intracellularly [94]– which may act as a source of foreign epitopes when exposed by maternal antigen-presenting cells. In addition, HLA genotyping studies in oocyte donation pregnancies revealed that the level of HLA matching between the mother and child in multiple alleles was positively associated with successful, uncomplicated pregnancies [95, 96]. This suggests maternal immune exposure to more than just placental cytotrophoblast cells, which is where fetal microchimeric cells transferred to maternal circulation during pregnancy might come in.

As for the maternal immune response resulting in tolerance to fetal alloantigens, Tregs (Regulatory T cells), which are a subset of T cells involved in immune regulation and self-recognition [97], appear to play an important role [98, 99]. In mice, depletion of maternal Tregs promotes rejection of the conceptus and reproductive failure [99, 100]. In human pregnancy, reduced total numbers of maternal Tregs in maternal circulation and decidua basalis are associated with preeclampsia [101] and miscarriage [102]. Interestingly, most Tregs in the decidua are not fetus-specific [103] and while miscarriage is associated with lower overall Treg cell numbers, and thereby a reduction in the absolute number of fetus-specific maternal Tregs, the proportion of clonally expanded fetus-specific Tregs is similar to what is observed in healthy, successful pregnancies [104]. In contrast, preeclampsia is associated with reduced clonal expansion of fetus-specific Tregs relative to the total number of decidual Tregs [104]. It has been proposed that the observed decrease in maternal Tregs in preeclampsia may be due to them having a senescent phenotype associated with apoptosis [105, 106], poor proliferative potential and functional impairment [107, 108]. Hypothetically, the maternal immune system would be less equipped to downregulate a fetus-specific maternal effector cell response to the increased amounts of circulating fetal microchimerism associated with preeclampsia.

The fetal immune system is also exposed to allogeneic *maternal* antigens during pregnancy. Though increased maternal microchimerism in the setting of preterm labor was associated with an increase in fetal central memory T cells (a subtype of effector T cells), the levels of fetal Tregs remained the same [74], suggesting that fetal tolerance to non-inherited alleles may be acquired even in preterm labor as has been shown in healthy pregnancies. Specifically, the fetal Tregs that mediate tolerance towards non-inherited maternal antigens are generated when maternal cells cross the placenta and home to fetal lymph nodes (Fig. 3) [109]. However, like other CD4 + T cells, Tregs require low-level antigen stimulation for long-term persistence [110], and it has been proposed that this is achieved through the presence of maternal microchimeric cells residing in immune tissues [109]. This is in line with the findings of higher success rates in solid organ [111] and bone marrow [112] transplantation when the donor alloantigens and non-inherited maternal alleles overlap. Furthermore, maintained tolerance to non-inherited maternal alleles may partly explain the observed protection against preeclampsia associated with maternal proband microchimerism during pregnancy [29]. Mouse studies by Sing Sing Way’s group have further expanded our understanding of the role of maternal microchimerism in pregnancy [10, 11, 92]. During allogeneic mating, the proband’s Tregs protect against fetal wastage when non-inherited maternal antigens and fetal alloantigens matched (Fig. 3) [11]. Experimental antibody-induced depletion of maternal microchimeric cells from the proband prior to mating reverted this protective effect, highlighting the importance of low-level antigenic stimulation in sustaining these specific Tregs. Mother of the proband microchimeric cells sequestered within the proband’s immune cell niches may thus facilitate pregnancies where inherited paternal alleles in the fetus overlap with or resemble non-inherited maternal alleles expressed on mother of the proband microchimeric cells.

Mother of the proband mirochimeric cells are depleted naturally during pregnancy, displaced by fetal microchimeric cells [10, 113]. The exact mechanism of displacement is unknown, but has been observed in both humans and animals. In women, circulating mother of the proband microchimerism was negatively associated with parity [113]. This is clearly demonstrated in mice, where pregnancy displaces mother of the proband microchimerism and thereby also erases the proband’s tolerance to non-inherited maternal alleles in subsequent pregnancies [10]. Fetal microchimerism, on the other hand, was not associated with parity in multiparous women [113]. The latter observation supports the concept that newly acquired microchimerism displaces previous microchimerism residing in stem cell and immune cell niches. In other words, fetal cells from multiple pregnancies do not seem to accumulate, but rather to compete for a restricted capacity. It is difficult to assess whether birth order determines which fetal cell population will be dominant in multiparous women. In a study by Gammill et al., only four families met the criteria to evaluate fetal microchimerism from sequential pregnancies. Two of these women had measurable circulating microchimerism, and in both cases, these cells stemmed from the second pregnancy [113]. Although this sample is too small to draw any conclusions, the findings do not contradict the observations in mice, where fetal cells from subsequent pregnancies outcompete cells from previous pregnancies [10].

Interestingly, displacement of fetal microchimerism in mice does not erase the protective effect of previous pregnancies with overlapping alloantigens [10]. The persistent immunotolerance towards previously encountered fetal alloantigens in the apparent absence of relevant fetal microchimerism and the associated Tregs is attributable to a particular subset of T cells termed exTregs. These exTregs have previously been implicated in autoimmune disorders, as the differentiation from Tregs to exTregs tends to be accompanied by a more proinflammatory phenotype [114]. However, in the context of pregnancy, exTregs may apparently be of benefit, involved in reproductive fitness. Taken together, the presence of fetal microchimerism sustaining Tregs, or the presence of exTregs in the absence of fetal microchimerism, may account for some of the protective effect of parity against preeclampsia and other pregnancy disorders.

## Future perspectives/conclusion

Here, we have reviewed the current literature on microchimerism in healthy pregnancies and pregnancy complications related to placental dysfunction. The quantity of circulating microchimerism varies by source and cellular phenotype of the microchimeric cells across the various pregnancy complications, suggesting that these cells may be impacted by or play a role in their underlying pathophysiology.

Based on ours and other findings, we have proposed that fetal microchimerism is associated with both stages of the two-stage model of preeclampsia, i.e. placental dysfunction and maternal endothelial dysfunction [30]. Meanwhile, mother of the proband microchimerism has been postulated to contribute beneficially to placentation as it is negatively correlated with miscarriage and preeclampsia [29, 73]. Interestingly, maternal microchimerism in fetal circulation is elevated in preterm labor, and has been proposed to play a role in initiating uterine contractions [74]. These hypotheses remain to be investigated.

One of the fetal origin cell types that have been detected in fetal microchimerism are mesenchymal stem cells [76, 77]. They affect trophoblast function in vitro and might thereby impact plantation [83, 84]. Investigation of fetal mesenchymal stem cells in decidua basalis in parallel with extravillous trophoblasts could shed some light on possible mechanisms underlying the evolutionary benefit of microchimerism. Furthermore, their function as signalling cells could have implications in maternal tissues as well. Finally, fetal mesenchymal stem cells display a senescent phenotype during preeclampsia [85, 86], which might have an inflammatory effect locally in the placenta as well as in the maternal periphery.

Animal studies reveal the involvement of microchimerism in immunological tolerization processes [10, 11, 92]. This has yet to be demonstrated in humans, but is in line with the observation of increased mother of the proband microchimerism in the circulation of healthy pregnancies [29]. Thorough study of HLA genotypes in three generations (grandmother, mother and offspring) with longitudinal follow-up for multiple pregnancies, combined with fetal and maternal microchimerism quantification and anti-body measurements could shed some light on this issue, but is technically demanding in humans.

The field of microchimerism challenges our understanding of transgenerational health and disease development. Our studies point to the placenta likely representing the crucial organ for orchestrating microchimeric processes. Our future research plan involves exploring potential avenues of fetal cell transfer during normal pregnancies and preeclampsia, as well as comparing fetal microchimeric cell properties in these two clinical settings. We also aim to assess if maternal biomarkers of cardiovascular health and inflammation correlate with fetal microchimerism quantity in maternal circulation across pregnancy outcome groups, both during and after pregnancy, and how these associations may be influenced by fetal-maternal histocompatibility [115]. We believe further studies aimed at elucidating the mechanistic pathways underlying cross-placental cell transfer and the intrinsic properties of fetal and maternal microchimerism could be instrumental in the advancement of female reproductive and cardiovascular health.

## Electronic supplementary material

Below is the link to the electronic supplementary material.

Supplementary Material 1

Supplementary Material 2

Supplementary Material 3

## Data Availability

Data sharing not applicable to this article as no datasets were generated or analysed during the current study.
